# Conductometric and Thermodynamic Studies of Selected Imidazolium Chloride Ionic Liquids in N,N-Dimethylformamide at Temperatures from 278.15 to 313.15 K

**DOI:** 10.3390/molecules29061371

**Published:** 2024-03-19

**Authors:** Zdzisław Kinart

**Affiliations:** Department of Physical Chemistry, Faculty of Chemistry, University of Lodz, Pomorska 163/165, 90–236 Lodz, Poland; zdzislaw.kinart@chemia.uni.lodz.pl

**Keywords:** electric conductivities, ionic liquids, N,N-Dimethylformamide, thermodynamic function

## Abstract

This scientific article presents research on the electrical conductivity of imidazole-derived ionic liquids (1-methylimidazolium chloride, 1-ethyl-3-methylimidazolium chloride, 1-butyl-3-methylimidazolium chloride, 1-hexyl-3-methylimidazolium chloride and 1-methyl-3-octylimidazolium chloride) in the temperature range of 278.15–313.15 K in N,N-Dimethylformamide. The measurement methods employed relied mainly on conductometric measurements, enabling precise monitoring of the conductivity changes as a function of temperature. Experiments were conducted at various temperature values, which provided a comprehensive picture of the conducting properties of the investigated ionic liquids. The focus of the study was the analysis of the conductometric results, which were used to determine the conductivity function as a function of temperature. Based on the obtained data, a detailed analysis of association constants (K_A_) and thermodynamic parameters such as enthalpy (∆H^0^), entropy (∆S^0^), Gibbs free energy (∆G^0^), Eyring activation enthalpy for charge transport ($\Delta H_{\lambda }^{‡}$) and diffusion processes (D^0^) was carried out. The conductometric method proved to be an extremely effective tool for accurately determining these parameters, significantly contributing to the understanding of the properties of imidazole-derived ionic liquids in the investigated temperature range. As a result, the obtained results not only provide new insights into the electrical conductivity of the studied ionic liquids but also broaden our knowledge of their thermodynamic behavior under different temperature conditions. These studies may have significant implications for the field of ionic liquid chemistry and may be applied in the design of modern materials with desired conducting properties.

## 1. Introduction

Ionic liquids, because of their unique physicochemical properties, currently constitute an area of intense scientific research. One of the intriguing research aspects is the electrical conductivity of ionic liquids, closely associated with their structure and the dynamic nature of ions [1,2,3,4,5]. In this context, ionic liquids based on imidazole derivatives represent a particularly interesting group of compounds, given their diverse applications that range from electrochemistry to the pharmaceutical industry. The current interest in ionic liquids is mainly based on their use as solvents or catalysts in various reactions [6,7,8,9,10,11,12,13,14].

Ionic liquids represent a fascinating area of scientific research due to their exceptional physicochemical properties, which are applicable in various fields, from electrochemistry to the pharmaceutical industry [15,16,17,18,19,20,21,22,23,24], and different applications such as solvents or catalysts in various reactions [6,7,8,9,10,11,12,13,14]. In this context, ionic liquids based on imidazole derivatives represent a particularly interesting group of compounds, given their diverse applications [25]. Consequently, imidazole-derived ionic liquids have been at the forefront of interest, and their properties in N,N-Dimethylformamide (DMF) have become the subject of intensive investigation.

Research on the properties of imidazole-derived ionic liquids in DMF focuses on various aspects, including electrical conductivity, molecular structure and interactions with the solvent environment [26,27,28,29]. Conductometric measurements conducted in this environment enable the precise determination of the changes in conductivity with temperature, providing crucial insights into the dynamics of ionic liquids. Additionally, investigations into the properties of ionic liquids in DMF yield essential data regarding their thermal stability, crucial for potential practical applications. These properties are key to the development of modern technologies, such as electrochemical energy storage devices or materials with advanced conducting properties.

In summary, research on imidazole-derived ionic liquids in the environment of N,N-Dimethylformamide opens new perspectives to understand their properties and potential applications in various scientific and industrial fields.

A review of the literature indicates that the electrical conductivity of electrolytes in DMF as a function of temperature has not previously been studied using imidazole-derived ionic liquids. However, the literature provides data on the physical properties of pure ionic liquids. Some studies report molar conductivity data for pure ionic liquids or two-component IL mixtures with various solvents [30,31,32,33,34,35,36,37,38,39,40].

In this article, we focus on investigating the electrical conductivity of imidazole-derived ionic liquids over a wide temperature range, ranging from 278.15 K to 313.15 K. Electrical conductivity measurements were performed using conductometric techniques, enabling the precise determination of changes in conductivity properties as a function of temperature.

The goal of our research is not only to provide new data regarding the electrical conductivity of imidazole-derived ionic liquids but also to deepen our understanding of their thermodynamic behavior in the context of temperature variations. The conductometric method employed in our study allows the determination of the conductivity function, which is a crucial step in analyzing the properties of these liquids under different conditions.

The presented results have potential applications in the advancement of new electrochemical technologies and in the design of materials with controlled conducting properties. Furthermore, understanding the thermodynamic behavior of imidazole-derived ionic liquids may contribute to the improvement of the industrial processes in which these compounds find applications.

## 2. Results and Discussion

The density, viscosity and relative permittivity values for N,N-Dimethylformamide necessary to calculate the limiting conductivity and association constant values are compiled in Table 1. The values of the dielectric constant were obtained from the literature [40].

To convert molonity ($\tilde{m}$) into molarity (c), the values of density gradients (b) were determined independently and used in the following equation:

(1) $$c\tilde{m}=q=q_{0}+b\cdot \tilde{m}$$

where q is the density of the solution.

Molarity (c) was needed for the conductivity equation. The density gradients and the molar conductivity of the imidazolium salts (Λ_m_) are presented in Table 2 as a function of molality and are visible in Figures S1–S5.

As evident, the molar conductivity values exhibit a linear trend with respect to concentration. These values increase with temperature but also decrease with the rise in the molecular weight of the investigated ionic liquid.

The conductance data were analyzed using the Fuoss–Justice equation [42,43], following the low-concentration chemical model (lcCM) used for the electrical conductivity calculations [44], applying the following equations:

(2) $${\;\Lambda }_{m}=\alpha [\Lambda^{o}-S{\left(\alpha c\right)}^{\frac{1}{2}}+E\left(\alpha c\right)\ln\left(\alpha c\right)+J\left(\alpha c\right)-J_{\frac{3}{2}}{\left(\alpha c\right)}^{\frac{3}{2}}$$

(3) $$\;K_{A}=\frac{1-\alpha }{\alpha^{2}cy_{\pm }^{2}}$$

and

(4) $$\ln y_{\pm }=-\frac{{A\alpha }^{1/2}c^{1/2}}{1+{\mathrm{Br}\alpha }^{1/2}c^{1/2}}$$

In these equations, Λ^o^ is the limiting molar conductivity, α is the degree of electrolyte dissociation, K_A_ is the constant of ionic association, R is the ion distance parameter [45], y_±_ is the ion activity on a molar scale, and A and B are the coefficients of the Barethe–Hückel equation. The analytical form of the parameters S, E, J and J_3/2_ is presented in works [46,47,48]. The values of Λ^o^, K_A_ and R were obtained using the well-known procedure provided by Fuoss [42] and are presented in Table 3. As indicated in Table 3, the association constants are practically negligible, suggesting that these electrolytes exist predominantly as free ions in DMF.

**Figure 1:**
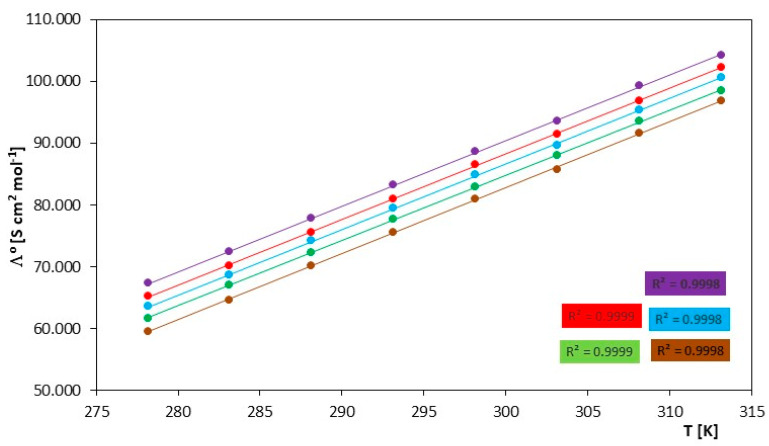
Temperature dependence of limiting molar conductances, (Λ^o^), for investigated ILs in N,N-Dimethylformamide, for IL: (⬤) [mim][Cl], (⬤) [emim][Cl], (⬤) [bmim][Cl], (⬤) [hmim][Cl] and (⬤) [omim][Cl].

The molar conductivity values presented in Figures S1–S5 exhibit a linear trend, decreasing as the concentration increases. Analyzing the limiting molar conductivity values presented in Table 3 and Figure 1, these values increase with increasing temperature and also with the increasing molecular weight of the investigated ionic liquid. This is consistent with the assumptions of the theory of molar conductivity. The increase in temperature is responsible for the enhanced mobility of free ions. It is observed that the limiting molar conductivity values decrease with the elongation of the chain length of the investigated ionic liquids but also increase with temperature within a single ionic liquid.

**Figure 2:**
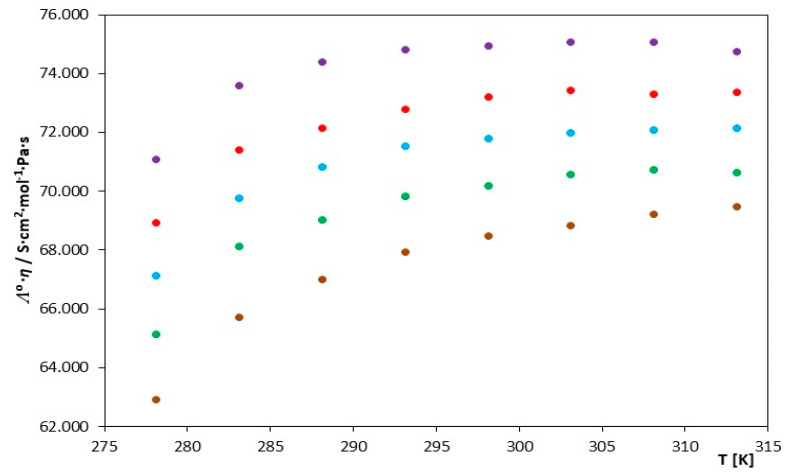
The course of changes in the value of the Walden product as a function of temperature for the investigated ILs in N,N-Dimethylformamide, for IL: (⬤) [mim][Cl], (⬤) [emim][Cl], (⬤) [bmim][Cl], (⬤) [hmim][Cl] and (⬤) [omim][Cl].

The analysis of the parameters of the Walden product parameters (Λ₀·η) in Table 3 and Figure 2 reveals that within the temperature range of 278.15–295.15 K, these values show an increasing trend. However, after exceeding the temperature of 295.15 K, these values stabilize, suggesting that the mobility is influenced by the viscosity of the solvent itself, namely DMF, in this case. This implies that the discussed imidazole-derived ionic liquids are minimally solvated by the molecules of the solvent. Similar properties were observed when other solvents used these ionic liquids [49,50].

**Figure 3:**
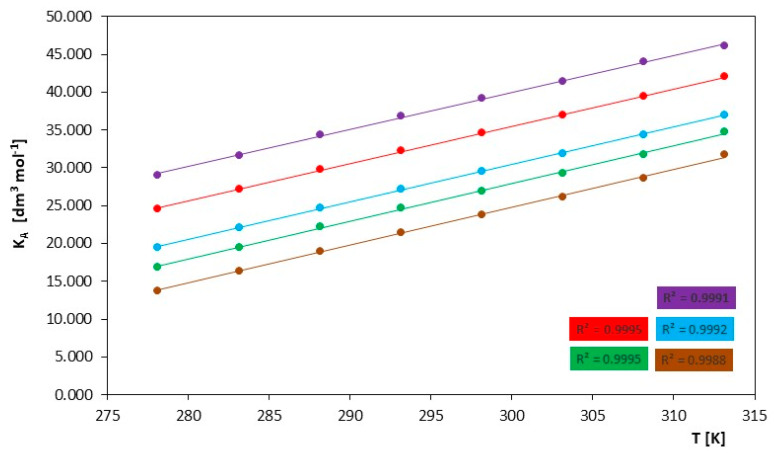
Course of changes in association constants, (K_A_), as a function of temperature for the investigated ILs in N,N-Dimethylformamide, for IL: (⬤) [mim][Cl], (⬤) [emim][Cl], (⬤) [bmim][Cl], (⬤) [hmim][Cl] and (⬤) [omim][Cl].

The analysis of the values of the association constant (K_A_) in Table 3 and Figure 3 indicates that the association constant increases with temperature. This suggests that at higher temperatures, the discussed ionic liquids have an enhanced ability to form associative compounds. The increase in the association constant may affect the stability of the ions, which, in turn, can affect their conductivity. The increase in K_A_ influences the dielectric properties of the liquid, which, in turn, may translate into electrical conductivity values.

An increase in the association constant is associated with a substance’s greater ability to form associative compounds, influencing its structure and intermolecular interactions. Associative compounds can lead to increased molecular polarizability, affecting the dielectric constant [51,52].

The dielectric properties of a substance directly affect its ability to conduct electricity. An increase in the dielectric constant typically corresponds to better insulating properties (lower electrical conductivity). In the case of ionic liquids, a high dielectric constant may favor ion solvation and increase ion stability in the solution, affecting their conductivity. An increase in the association constant may lead to the formation of larger associative structures, which affects the mobility of ions. An enhanced ability to form associative compounds may also influence the equilibrium between ions and their associative structures, likely affecting electrical conductivity. Comparison with the literature values from previous works in the studied solvent with the discussed ionic liquids could not be made because these are the first works presented in the literature.

Using temperature measurements of electrical conductivity, we were able to determine the activation enthalpy of Eyring for charge transport.

(5) $$ln\Lambda_{0}+2/3lnd_{0}=-\frac{\Delta H_{\lambda }^{‡}}{R\cdot T}+B$$

where B is an empirical constant.

**Figure 4:**
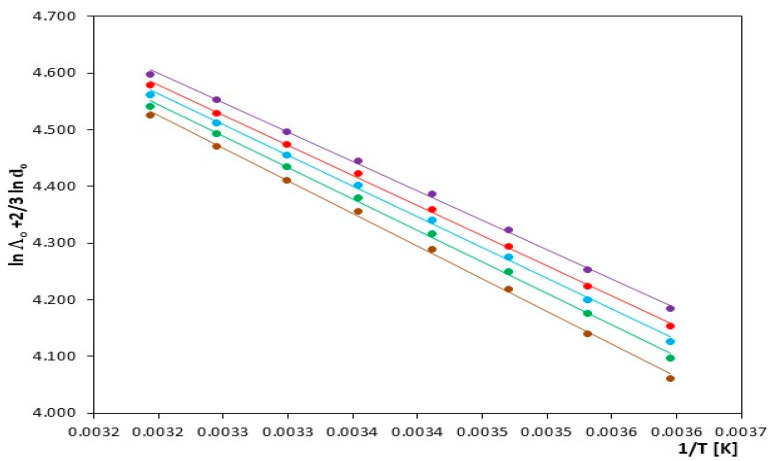
Plot of ln Λ_o_ + 2/3 lnd_o_ as a function of 1/T in N,N-Dimethylformamide, for IL: (⬤) [mim][Cl], (⬤) [emim][Cl], (⬤) [bmim][Cl], (⬤) [hmim][Cl] and (⬤) [omim][Cl].

The values of $\Delta H_{\lambda }^{‡}$ were obtained from the slope of the linear function $ln\Lambda_{0}+2/3lnd_{0}$ as a function of 1/T [K], as shown in Figure 4. It is evident that these values align linearly with a very high linear correlation close to unity.

The values of $\Delta H_{\lambda }^{‡}$ for the investigated ionic liquids in N,N-Dimethylformamide are shown in Table 4.

They follow the order mim < emim < bmim < hmim < omim. For [mim], the $\Delta H_{\lambda }^{‡}$ value is the smallest. In contrast, for omim, the enthalpy of charge transfer is the largest. This result is due to the presence of a larger substituent in the [omim]^+^ cation compared to that in the [emim]^+^ cation. The opposite trend is observed when the diffusion coefficient values for the investigated ionic liquids are calculated. In this study, it was possible to estimate the diffusion coefficient values using the Nernst–Hartley relationship [53]:

(6) $$\;D^{0}=\frac{R\cdot T\cdot \Lambda^{o}}{F^{2}}$$

where R is the gas constant, and F is the Faraday constant.

**Figure 5:**
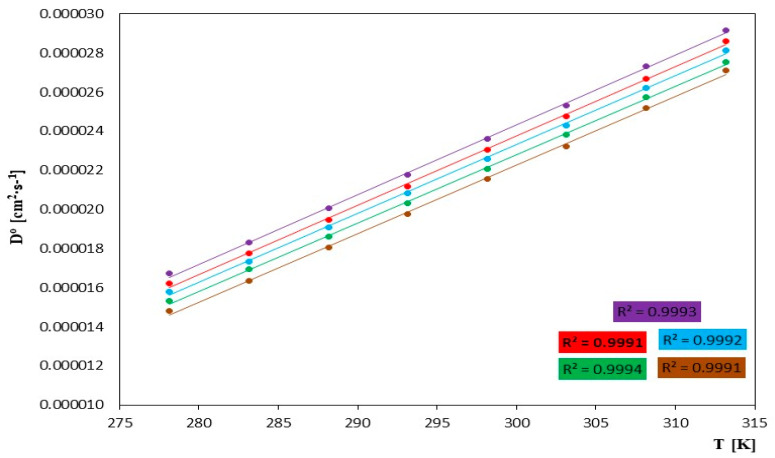
The course of changes in the value of the diffusion coefficient D^0^ [cm^2^∙s^−1^] as a function of temperature *T* [K] for the tested ionic liquids in N,N-Dimethylformamide, for IL: (⬤) [mim][Cl], (⬤) [emim][Cl], (⬤) [bmim][Cl], (⬤) [hmim][Cl] and (⬤) [omim][Cl].

These D^0^ values decrease in the order mim > emim > bmim > hmim > omim and increase with an increase in temperature for a given ionic liquid. The decreases are attributed to the increase in the molecular weight of the investigated ionic liquid, as observed in Table 5 and Figure 5. An increase in the molecular weight of ionic liquid molecules generally leads to a slowing down of the diffusion process. This happens because molecules with greater mass face more difficulty in moving within the environment due to their mass and inertia. An increase in molecular weight can also lead to an increase in the viscosity of the ionic liquid, making diffusion more challenging.

The diffusion rates increase with an increase in temperature, confirming the validity of the described relationship. The temperature significantly influences the diffusion process of ionic liquids. Changes in temperature affect the rate of this process and can also affect other properties of ionic liquids. An increase in temperature usually enhances the average kinetic energy of the ionic liquid molecules, accelerating their thermal motions and leading to faster diffusion. In practice, according to the Arrhenius equations, the diffusion rate is proportional to the exponential function of the temperature. Temperature can affect the viscosity of the liquid, which in turn influences the diffusion process. An increase in temperature typically results in a decrease in the viscosity of the liquid, facilitating molecular movement and increasing mobility.

The temperature dependence of the association constant was used to calculate the Gibbs free energy, ΔG^0^ [38]:

ΔG^0^(T) = −R T lnKA(T) (7)

ΔG^0^(T) can also be expressed by the polynomial equation

ΔG^0^(T) = A + B T + C T2 (8)

The entropy, ΔS^0^, and enthalpy, ΔH^0^, of ion association are defined as

(9) $$\Delta S^{0}\left(T\right)=-{\left(\frac{\delta \Delta G^{0}}{\delta T}\right)}_{p}=-B-2CT$$

ΔH^0^ = ΔG^0^ + T ΔS^0^ (10)

**Figure 6:**
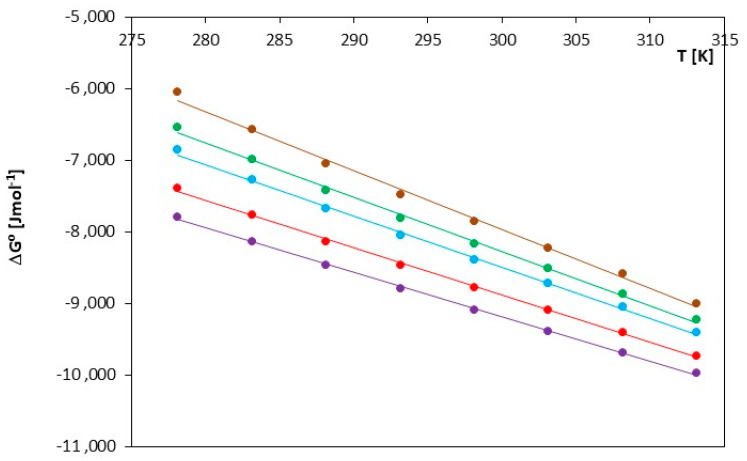
Changes in the value of Gibbs free energy, ΔG^0^, for the investigated ILs in N,N-Dimethylformamide, for IL: (⬤) [mim][Cl], (⬤) [emim][Cl], (⬤) [bmim][Cl], (⬤) [hmim][Cl] and (⬤) [omim][Cl].

**Figure 7:**
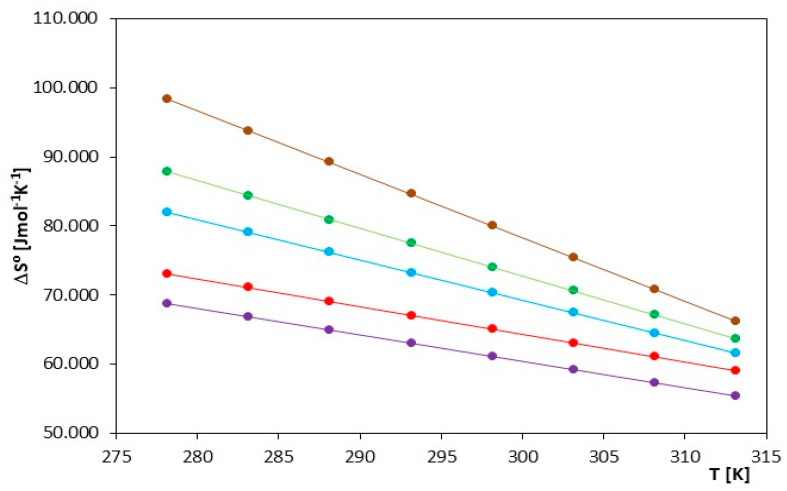
Changes in the value of the entropy of ion association, ΔS^0^, for the investigated ILs in N,N-Dimethylformamide, for IL: (⬤) [mim][Cl], (⬤) [emim][Cl], (⬤) [bmim][Cl], (⬤) [hmim][Cl] and (⬤) [omim][Cl].

**Figure 8:**
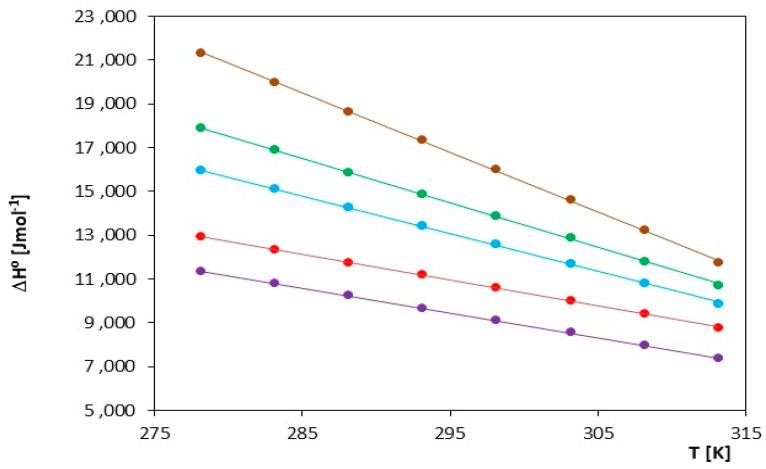
Changes in the value of the enthalpy of ion association, ΔH^0^, for the investigated ILs in N,N-Dimethylformamide, for IL: (⬤) [mim][Cl], (⬤) [emim][Cl], (⬤) [bmim][Cl], (⬤) [hmim][Cl] and (⬤) [omim][Cl].

The thermodynamic functions described above (ΔG^0^, ΔS^0^, ΔH^0^) were measured at the temperature range T = (278.15 K–313.15 K) and are presented in Table 6 and Figure 6, Figure 7, Figure 8.

The thermodynamic values ΔG^0^ presented in Table 6 and Figure 6 indicate that the spontaneity of ionic pair formation is greater for ionic liquids containing a smaller cation, i.e., [mim]. An increase in temperature leads to a higher number of negative ΔG^0^ values, signifying a shift in thermodynamic equilibrium toward the formation of ionic pairs. As seen in Figure 7 and Figure 8, both the association entropy and the enthalpy values increase with increasing temperature for both investigated electrolytes.

ΔG^0^, ΔH^0^ and ΔS^0^ decrease with increasing temperature. This indicates that, for this ionic liquid, the reaction becomes more thermodynamically favorable at higher temperatures. The decrease in ΔH^0^ suggests that the ionization process is more endothermic at higher temperatures, and the decrease in ΔS^0^ indicates greater entropy ordering, as shown in Figure 7 and Figure 8 and Table 6. The reduction in ΔG^0^ values may indicate an increased spontaneity of the ionization process at higher temperatures.

In general, for all investigated ionic liquids, it can be observed that the ionization process becomes more thermodynamically favorable at higher temperatures, which may be significant in applications where temperature control is crucial for their properties. Positive values of ΔH^0^ indicate that the ion vaporization process is endothermic. At a temperature of 278.15 K, this process is more endothermic for [mim][Cl], while at a temperature of 318.15 K, it is more endothermic for [omim] [Cl]. From Equation (7), the results show that entropic effects seem to dominate over enthalpic effects, as the Gibbs free energy (ΔG^0^) is negative, indicating that the formation of ionic pairs is exothermic in both cases.

## 3. Materials and Methods

### 3.1. Materials

Conductometric measurements were performed using five ionic liquids: 1-methylimidazolium chloride, 1-ethyl-3-methylimidazolium chloride, 1-butyl-3-methylimidazolium chloride, 1-hexyl-3-methylimidazolium chloride and 1-methyl-3-octylimidazolium chloride. N,N-Dimethylformamide was employed as the solvent. All reagents used were of high purity. All the necessary details can be found in Table 7.

### 3.2. Conductometric Measurements

All solutions were prepared using an analytical balance (Sartorius RC 210D)(Goettingen, Germany) with an accuracy of ±1·10^−5^ g. The measurement procedure was based on the method described by Bešter-Rogač et al. [54,55,56]. Electrical conductivity measurements were performed using a three-electrode cell constructed with PYREX glass and a Precise Component Analyzer bridge type 6430B (Wayne-Kerr, West Sussex, UK). The measurements were made at various frequencies, ν, (0.2, 0.5, 1, 2, 3, 5, 10 and 20) kHz. The temperature was maintained within 0.003 K using a calibrated UltraUB 20F with a circulating cooler DLK 25, Lauda, (Lauda-Königshofen, Germany) The experimental procedure for conductometric measurements is detailed in the literature [57,58,59]. The three-electrode conductometric cell was calibrated at each temperature using aqueous KCl solutions [60]. All measured conductivity values, λ = 1/R_∞_, resulted from extrapolating the cell resistance, R(ν), to infinite frequency, R_∞_ = lim_ν→∞_R(ν), using the empirical function R(ν) = R_∞_ + A/ν. All data were corrected for the specific conductivity of the solvent. Densities were measured using an Anton Paar DSA 5000M (Graz, Austria) equipped with a thermostat with temperature stability within ±0.001 K. The densimeter was calibrated using extra-pure water, previously degassed ultrasonically [61,62]. Viscosities were measured with a Viscometer AVS 350 (Schott Geräte, Mainz, Germany). The flow time of the liquid in the Ubbelohde capillary viscometer of the same company was optoelectronically recorded with an accuracy of 0.01 s [63]. The viscometer with the measurement stand was immersed in a water-filled thermostat. The temperature was controlled by a Circulator DC 30 thermostat head (HAKE, Bremerhaven, Germany). The temperature was maintained with a Julabo F32 precision thermostat (Julabo Labortechnik GmbH, Seelbach, Germany). The temperature control accuracy was 0.01 K. The error in the relative viscosity was estimated at 0.01%.

## 4. Conclusions

The molar conductivity of the ionic liquid solutions, derivatives of imidazole in DMF, was provided at temperatures ranging from 278.15 K to 313.15 K. Conductivity data were analyzed using the Fuoss–Justice equation. It was observed that the limiting molar conductivity values increase with temperature but exhibit an inverse trend because of the elongation of the alkyl chain in the investigated ionic liquid. The association constants increase with temperature (as the relative permeability of the solvent decreases), but, similar to electrical conductivity, they decrease with increasing the alkyl chain length of the IL. The determined values of the Walden products for the discussed imidazole-derived ionic liquids in N,N-Dimethylformamide illustrate the influence of viscosity on associative-solvation effects. On the basis of these values, one can infer how the diffusion phenomenon occurs for the analyzed ionic liquids. Conductometric measurements were used to determine and analyze thermodynamic functions such as ΔG^0^, ΔH^0^ and ΔS^0^. The ΔH^0^ values are positive, suggesting that the process of ion pair formation is endothermic. Negative values of the Gibbs free energy indicate the predominance of entropic effects over enthalpic effects during the analysis of the behavior of ionic liquids in N,N-Dimethylformamide.

The results obtained can have practical applications in the context of sustainable development in several aspects.

First, data regarding the conductivity of ionic liquids can be utilized in the development of more efficient industrial processes, especially in the separation and processing of chemical substances. Optimizing these processes based on the obtained results can help minimize energy consumption and reduce the emission of harmful substances into the environment.

Second, thermodynamic results can be employed in designing more sustainable chemical processes, as they allow a better understanding of the thermodynamic behaviors and phase equilibria in a given system. The unique properties of ionic liquids identified in the research can serve as a basis for the development of new, more environmentally friendly solutions in the chemical industry.

Additionally, in the context of sustainable development, the data obtained can support research on the possibilities of recycling and reusing imidazolium chloride ionic liquids. Understanding their behaviors under different temperature conditions can lead to the development of effective methods for the recovery and regeneration of these ionic liquids, contributing to the reduction in chemical waste.

## Figures and Tables

**Table 1:** Density, (*d*), viscosity (*η*) and relative permittivity, (*ε*_r_) [41], for N,N-Dimethylformamide in the temperature range from *T* = (278.15 to 313.15) K at *p* = 0.1 MPa ^a^.

278.15 K	283.15 K	288.15 K	293.15 K	298.15 K	303.15 K	308.15 K	313.15 K
d/g·cm^−3^							
0.962455	0.958092	0.953299	0.948555	0.943802	0.939051	0.934226	0.929441
η/mPa·s							
1.0555	1.0158	0.9545	0.8985	0.8455	0.7990	0.7553	0.7172
ε_r_							
40.88	39.61	38.68	37.75	36.81	35.88	34.95	34.01

^a^ Standard uncertainties *u* are *u*(*p*) = 0.05 p and *u*(T) = 0.01 K, and the combined expanded uncertainties U_c_ are U_c_(d_0_) = 2‧10^−5^ g‧cm^−3^ and U_c_(η*)* = 0.0030 mPa‧s (level of condidence = 0.95).

**Table 2:** Molar conductances, (Λ_m_), and corresponding molalites, (*m*), for studied ILs in N,N-Dimethylformamide over the temperature range from *T* = (278.15 to 313.15) K at pressure *p* = 101.3 kPa ^a^.

10^3^ m/ mol·kg^−1^	Λ_m_/S·cm^2^·mol^−1^							
[mim][Cl] + N,N-Dimethylformamide								
T/K	278.15	283.15	288.15	293.15	298.15	303.15	308.15	313.15
0.23745	67.945	73.150	78.654	83.105	88.957	93.938	99.771	104.637
0.56643	67.923	73.134	78.623	83.069	88.928	93.878	99.704	104.544
0.98884	67.909	73.119	78.588	83.041	88.877	93.832	99.641	104.472
2.22058	67.879	73.081	78.529	82.985	88.785	93.72	99.515	104.303
4.39830	67.842	73.035	78.462	82.898	88.649	93.569	99.325	104.052
7.93002	67.782	72.957	78.345	82.772	88.475	93.351	99.058	103.737
8.50124	67.777	72.945	78.331	82.76	88.454	93.314	99.026	103.682
12.6034	67.704	72.872	78.206	82.639	88.251	93.102	98.757	103.368
13.2076	67.704	72.858	78.192	82.615	88.228	93.028	98.687	103.293
14.0645	67.681	72.839	78.158	82.589	88.155	92.981	98.614	103.191
[emim][Cl] + N,N-Dimethylformamide								
0.22459	65.382	69.877	76.195	81.202	87.126	91.948	97.536	102.795
0.57245	65.358	69.846	76.136	81.139	87.067	91.850	97.439	102.656
0.98742	65.344	69.826	76.097	81.098	87.015	91.801	97.345	102.543
2.23547	65.303	69.775	76.023	81.018	86.892	91.664	97.156	102.342
4.25871	65.251	69.695	75.908	80.882	86.755	91.502	96.919	102.066
7.85241	65.175	69.608	75.747	80.689	86.523	91.264	96.583	101.629
8.51345	65.165	69.591	75.721	80.653	86.482	91.215	96.536	101.563
12.7134	65.081	69.488	75.528	80.434	86.264	90.922	96.152	101.122
13.1125	65.075	69.461	75.525	80.405	86.225	90.906	96.111	101.094
14.1542	65.035	69.421	75.471	80.376	86.158	90.774	96.003	100.925
[bmim][Cl] + N,N-Dimethylformamide								
0.21234	64.211	69.135	74.807	79.926	85.196	90.106	95.841	101.145
0.56154	64.179	69.106	74.755	79.878	85.094	89.992	95.765	100.954
0.99875	64.167	69.084	74.732	79.833	85.042	89.927	95.658	100.838
2.34211	64.107	69.011	74.638	79.739	84.892	89.758	95.452	100.558
4.42653	64.037	68.926	74.535	79.594	84.709	89.530	95.123	100.166
7.61321	63.957	68.828	74.415	79.436	84.496	89.265	94.772	99.749
8.62244	63.935	68.798	74.381	79.391	84.420	89.181	94.659	99.583
12.8198	63.863	68.695	74.209	79.177	84.074	88.835	94.204	98.919
13.2231	63.855	68.681	74.201	79.159	84.028	88.821	94.189	98.903
14.4331	63.838	68.646	74.159	79.108	83.937	88.691	94.019	98.758
[hmim][Cl] + N,N-Dimethylformamide								
0.20432	62.074	66.561	71.715	78.171	83.237	88.036	94.517	99.085
0.57245	62.063	66.536	71.675	78.137	83.169	87.945	94.421	98.923
0.98742	62.053	66.525	71.654	78.111	83.139	87.892	94.352	98.851
2.23547	62.028	66.489	71.595	78.046	83.033	87.786	94.221	98.674
4.25871	62.003	66.456	71.546	77.978	82.918	87.653	94.055	98.459
7.85241	61.946	66.387	71.441	77.871	82.727	87.402	93.764	98.098
8.51345	61.935	66.376	71.417	77.845	82.697	87.366	93.713	98.046
12.7134	61.884	66.301	71.301	77.725	82.457	87.102	93.403	97.649
13.1125	61.877	66.294	71.293	77.721	82.445	87.083	93.371	97.621
14.1542	61.859	66.276	71.256	77.681	82.353	87.016	93.317	97.491
[omim][Cl] + N,N-Dimethylformamide								
0.21254	59.885	64.032	69.789	74.902	80.161	85.184	91.037	96.146
0.56321	59.879	64.028	69.779	74.892	80.145	85.156	90.998	96.108
1.29652	59.871	64.016	69.765	74.868	80.124	85.113	90.943	96.038
2.32541	59.857	64.002	69.745	74.836	80.095	85.083	90.889	95.974
4.42515	59.843	63.982	69.716	74.809	80.048	85.017	90.821	95.879
7.63201	59.816	63.955	69.675	74.751	79.992	84.925	90.697	95.727
8.23521	59.812	63.949	69.672	74.745	79.984	84.912	90.686	95.692
12.9325	59.773	63.907	69.615	74.673	79.905	84.794	90.528	95.508
13.3252	59.771	63.905	69.615	74.671	79.90	84.788	90.506	95.498
14.8521	59.755	63.891	69.596	74.644	79.862	84.746	90.451	95.409

^a^ Standard uncertainties are *u*(*T*) = 0.01 K, *u*(*p*) = 0.05 MPa and *u*(*c*) = 10^−4^∙*c*, and the combined expanded uncertainty is *U*_c_(Λ) = 0.0005∙Λ (level of confidence = 0.95).

**Table 3:** Limiting molar conductances, (Λ^o^), association constants, (*K*_A_), and Walden products, (Λ^o^*η*), for studied ILs in N,N-Dimethylformamide in the temperature range from *T* = (278.15 to 313.15) K, with their standard error, (*σ*).

*T*/K	Λ^o/^S·cm^2^·mol^−1^	*K*_A/_dm^3^·mol^−1^	Λ^o^·*η*/S·cm^2^·mol^−1^·Pa·s	*R/nm*	*σ(*Λ*)*
[mim][Cl] + N,N-Dimethylformamide					
278.15	67.33 ± 0.01	29.07 ± 0.2	71.072	0.88	0.01
283.15	72.40 ± 0.01	31.68 ± 0.2	73.559	0.86	0.01
288.15	77.96 ± 0.02	34.36 ± 0.2	74.386	0.84	0.02
293.15	83.22 ± 0.01	36.84 ± 0.3	74.795	0.80	0.02
298.15	88.63 ± 0.02	39.13 ± 0.2	74.934	0.78	0.01
303.15	93.57 ± 0.02	41.47 ± 0.2	75.043	0.76	0.02
308.15	99.32 ± 0.01	43.96 ± 0.2	75.045	0.76	0.01
313.15	104.26 ± 0.01	46.07 ± 0.3	74.743	0.78	0.02
[emim][Cl] + N,N-Dimethylformamide					
278.15	65.28 ± 0.02	24.52 ± 0.2	68.908	0.86	0.01
283.15	70.25 ± 0.02	27.13 ± 0.2	71.375	0.82	0.01
288.15	75.61 ± 0.02	29.81 ± 0.1	72.144	0.80	0.01
293.15	80.97 ± 0.02	32.29 ± 0.1	72.773	0.78	0.01
298.15	86.58 ± 0.02	34.58 ± 0.2	73.200	0.76	0.02
303.15	91.52 ± 0.01	36.92 ± 0.1	73.399	0.75	0.02
308.15	96.97 ± 0.01	39.41 ± 0.1	73.269	0.76	0.02
313.15	102.31 ± 0.01	42.02 ± 0.1	73.346	0.78	0.01
[bmim][Cl] + N,N-Dimethylformamide					
278.15	63.58 ± 0.01	19.42 ± 0.1	67.113	0.86	0.01
283.15	68.65 ± 0.01	22.03 ± 0.2	69.749	0.76	0.02
288.15	74.21 ± 0.02	24.71 ± 0.2	70.808	0.72	0.02
293.15	79.57 ± 0.01	27.19 ± 0.2	71.514	0.66	0.02
298.15	84.88 ± 0.02	29.48 ± 0.2	71.763	0.64	0.01
303.15	89.72 ± 0.01	31.82 ± 0.3	71.956	0.65	0.02
308.15	95.37 ± 0.01	34.31 ± 0.2	72.060	0.71	0.01
313.15	100.61 ± 0.21	37.02 ± 0.2	72.127	0.77	0.03
[hmim][Cl] + N,N-Dimethylformamide					
278.15	61.68 ± 0.01	16.92 ± 0.1	65.108	0.88	0.01
283.15	67.05 ± 0.03	19.53 ± 0.2	68.124	0.76	0.01
288.15	72.31 ± 0.01	22.21 ±0.2	68.995	0.70	0.02
293.15	77.67 ± 0.02	24.69 ± 0.2	69.807	0.68	0.02
298.15	82.98 ± 0.01	26.98 ± 0.3	70.157	0.64	0.02
303.15	87.98 ± 0.01	29.32 ± 0.2	70.560	0.68	0.02
308.15	93.57 ± 0.02	31.81 ± 0.2	70.700	0.72	0.01
313.15	98.51 ± 0.02	34.72 ± 0.3	70.621	0.78	0.01
[omim][Cl] + N,N-Dimethylformamide					
278.15	59.58 ± 0.01	13.72 ± 0.2	62.891	0.88	0.01
283.15	64.65 ± 0.01	16.33 ± 0.2	65.685	0.74	0.01
288.15	70.21 ± 0.01	19.01 ± 0.3	66.991	0.68	0.02
293.15	75.57 ± 0.02	21.49 ± 0.2	67.919	0.66	0.02
298.15	80.98 ± 0.01	23.78 ± 0.2	68.466	0.62	0.01
303.15	85.82 ± 0.02	26.12 ± 0.2	68.828	0.70	0.02
308.15	91.57 ± 0.02	28.61 ± 0.2	69.189	0.78	0.02
313.15	96.91 ± 0.01	31.72 ± 0.2	69.474	0.81	0.01

**Table 4:** Transfer enthalpy values ($\Delta H_{\lambda }^{‡}$) for the investigated ionic liquids in the temperature range of 278.15 to 333.15 K.

$\Delta H_{\lambda }^{‡}$ **[J‧mol^−1^]**				
**[mim][Cl]**	**[emim][Cl]**	**[bmim][Cl]**	**[hmim][Cl]**	**[omim][Cl]**
8589	8838	9002	9189	9566

**Table 5:** The values of diffusion coefficient for ionic liquids, *D*^0^ [cm^2^·s^−1^], in N,N-Dimethylformamide over the temperature range from (278.15 to 313.15) K.

*D*^0^∙10^6^/cm^2^·s^−1^					
*T*/K	[mim][Cl]	[emim][Cl]	[bmim][Cl]	[hmim][Cl]	[omim][Cl]
278.15	16.721	16.212	15.790	15.318	14.797
283.15	18.303	17.759	17.355	16.950	16.344
288.15	20.055	19.451	19.091	18.602	18.062
293.15	21.780	21.191	20.825	20.327	19.778
298.15	23.591	23.046	22.593	22.087	21.555
303.15	25.325	24.770	24.283	23.812	23.227
308.15	27.324	26.678	26.237	25.742	25.192
313.15	29.149	28.604	28.128	27.541	27.094

**Table 6:** Standard thermodynamic quantities for the ion-association reaction for studied ILs in N,N-Dimethylformamide over the temperature range from *T* = (278.15 to 313.15) K.

*T*/K	ΔH^0^/J·mol^−1^	ΔG^0^/J·mol^−1^	ΔS^0^/J·mol^−1^·K^−1^
[mim][Cl] + N,N−Dimethylformamide			
278.15	11,340.96	−7792.58	68.79
283.15	10,800.18	−8135.06	66.87
288.15	10,244.55	−8473.26	64.96
293.15	9691.07	−8790.14	63.04
298.15	9135.92	−9089.56	61.13
303.15	8562.21	−9388.37	59.21
308.15	7963.94	−9692.61	57.30
313.15	7371.42	−9971.94	55.38
[emim][Cl] + N,N−Dimethylformamide			
278.15	12,929.64	−7398.94	73.08
283.15	12,355.37	−7770.07	71.08
288.15	11,769.26	−8132.96	69.07
293.15	11,190.07	−8468.85	67.06
298.15	10,612.40	−8783.14	65.05
303.15	10,016.62	−9095.46	63.04
308.15	9395.85	−9412.69	61.04
313.15	8752.55	−9732.37	59.03
[bmim][Cl] + N,N−Dimethylformamide			
278.15	15,949.93	−6859.69	82.00
283.15	15,112.71	−7279.86	79.08
288.15	14,262.86	−7683.44	76.16
293.15	13,420.96	−8049.87	73.24
298.15	12,578.53	−8387.61	70.32
303.15	11,711.45	−8720.79	67.40
308.15	10,811.49	−9057.64	64.48
313.15	9874.28	−9402.54	61.56
[hmim][Cl] + N,N−Dimethylformamide			
278.15	17,874.21	−6541.01	87.78
283.15	16,883.49	−6996.30	84.34
288.15	15,882.04	−7427.91	80.90
293.15	14,890.90	−7814.79	77.45
298.15	13,899.08	−8167.95	74.01
303.15	12,879.40	−8514.55	70.57
308.15	11,822.65	−8863.82	67.13
313.15	10,709.04	−9235.54	63.69
[omim][Cl] + N,N−Dimethylformamide			
278.15	21,315.01	−6056.20	98.40
283.15	19,986.85	−6575.04	93.81
288.15	18,651.40	−7055.19	89.21
293.15	17,328.86	−7476.48	84.62
298.15	16,003.12	−7854.99	80.02
303.15	14,641.67	−8223.28	75.42
308.15	13,233.62	−8592.19	70.83
313.15	11,740.45	−9000.26	66.23

**Table 7:** Structure and specification of used chemicals in this work.

Structure	Name, Abbreviation	CAS Number	Purity/%	Final Water Mass Fraction ^a^	Source
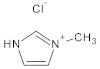	1-methylimidazolium chloride, [mim][Cl]	35487-17-3	98	0.00010	IoLiTec (Heilbronn, Germany)
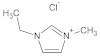	1-ethyl-3-methylimidazolium chloride, [emim][Cl]	65039-09-0	>98	0.0002	Sigma–Aldrich (Darmstadt, Germany)
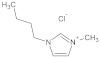	1-butyl-3-methylimidazolium chloride, [bmim][Cl]	79917-90-1	≥98	0.00013	Sigma–Aldrich (Darmstadt, Germany)
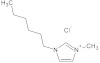	1-hexyl-3-methylimidazolium chloride, [hmim][Cl]	1142-20-7	≥98.5	0.0002	Sigma–Aldrich (Darmstadt, Germany)
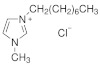	1-methyl-3-octylimidazolium chloride [omim][Cl]	64697-40-1	≥99	0.00012	IoLiTec (Heilbronn, Germany)
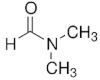	N,N-Dimethylformamide,	68-12-2	>99.8	0.00005	Sigma –Adrich (Darmstadt, Germany)

^a^ determined by Karl–Fischer titration.

## Data Availability

Data are contained within the article and Supplementary Materials.

## Supplementary Materials

The following supporting information can be downloaded at https://www.mdpi.com/article/10.3390/molecules29061371/s1, Figure S1. Temperature dependence of molar conductances, Λ_m_/S·cm^2^·mol^−1^ for [mim][Cl] in N,N-Dimethylformamide for (⬤) 278.15 K, (⬤) 283.15 K, (⬤) 288.15 K, (⬤) 293.15.15, K (⬤) 298.15 K, (⬤) 303.15 K, (⬤) 308.15 K and (⬤) 313.15 K; Figure S2. Temperature dependence of molar conductances, Λ_m_/S·cm^2^·mol^−1^ for [emim][Cl] in N,N-Dimethylformamide for (■) 278.15 K, (■) 283.15 K, (■) 288.15 K, (■) 293.15.15 K, (■) 298.15 K, (■) 303.15 K, (■) 308.15 K and (■) 313.15 K; Figure S3. Temperature dependence of molar conductances, Λ_m_/S·cm^2^·mol^−1^ for [bmim][Cl] in N,N-Dimethylformamide for (▲) 278.15 K, (▲) 283.15 K, (▲) 288.15 K, (▲) 293.15.15 K, (▲) 298.15 K, (▲) 303.15 K, (▲) 308.15 K and (▲) 13.15 K; Figure S4. Temperature dependence of molar conductances, Λ_m_/S·cm^2^·mol^−1^ for [hmim][Cl] in N,N-Dimethylformamide for (♦) 278.15 K, (♦) 283.15 K, (♦) 288.15 K, (♦) 293.15.15 K, (♦) 298.15 K, (♦) 303.15 K, (♦) 308.15 K and (♦) 313.15 K; Figure S5. Temperature dependence of molar conductances, Λ_m_/S·cm^2^·mol^−1^ for [omim][Cl*]* in N,N-Dimethylformamide for (✴) 278.15 K, (✴) 283.15 K, (✴) 288.15 K, (✴) 293.15.15 K, (✴) 298.15 K, (✴) 303.15 K, (✴) 308.15 K and (✴) 313.15 K.
