# Supplementary material for: Conductometric and Thermodynamic Studies of Selected Imidazolium Chloride Ionic Liquids in N,N-Dimethylformamide at Temperatures from 278.15 to 313.15 K
Source: Molecules. 2024 Mar 19;29(6):1371. doi: 10.3390/molecules29061371 (PMC10974909; doi:10.3390/molecules29061371)
Supplement: Supplementary file 1 [file molecules-29-01371-s001.zip › molecules-2915802-supplementary.pdf]

Conductometric and Thermodynamic Studies of Selected Imidazolium Chloride Ionic Liquids in N,N-Dimethylformamide at Temperatures from 278.15 to 313.15 K

Zdzisław Kinart*

University of Lodz, Faculty of Chemistry, Department of Physical Chemistry, 90 – 236 Lodz, Pomorska 163/165, Poland.
Correspondence: zdzislaw.kinart@chemia.uni.lodz.pl

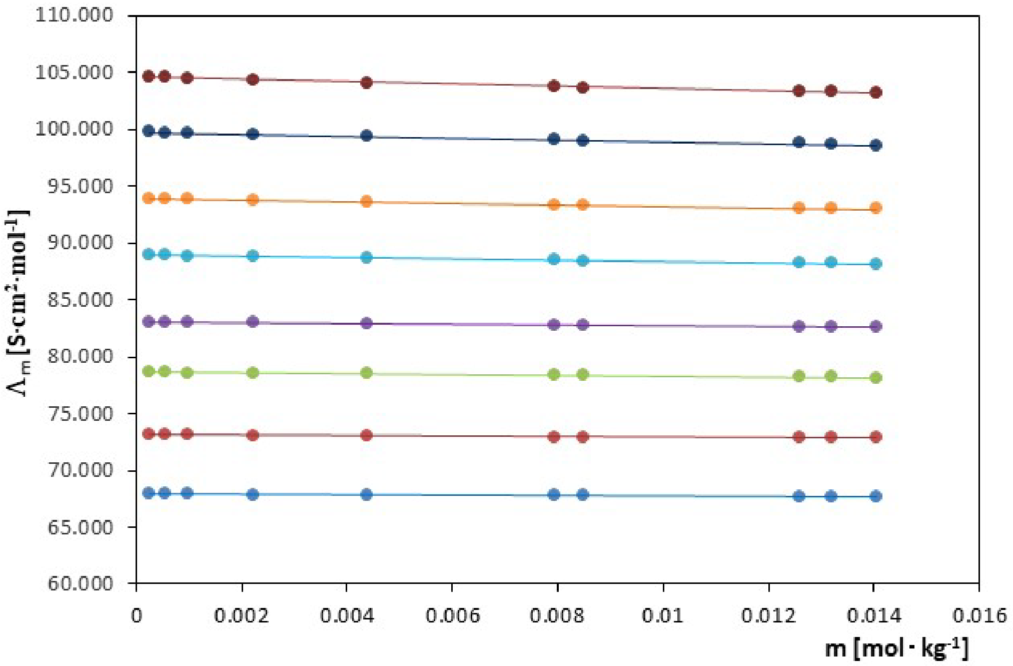

Figure S1. Temperature dependence of molar conductances, Λ_m / $S{\cdot}cm^2{\cdot}mol^{-1}$ for *[mim][Cl] in* N,N-Dimethylformamide for : (●) 278.15K, (●) 283.15K, (●) 288.15K (●) 293.15.15K (●) 298.15K, (●) 303.15K, (●) 308.15K and (●) 313.15K

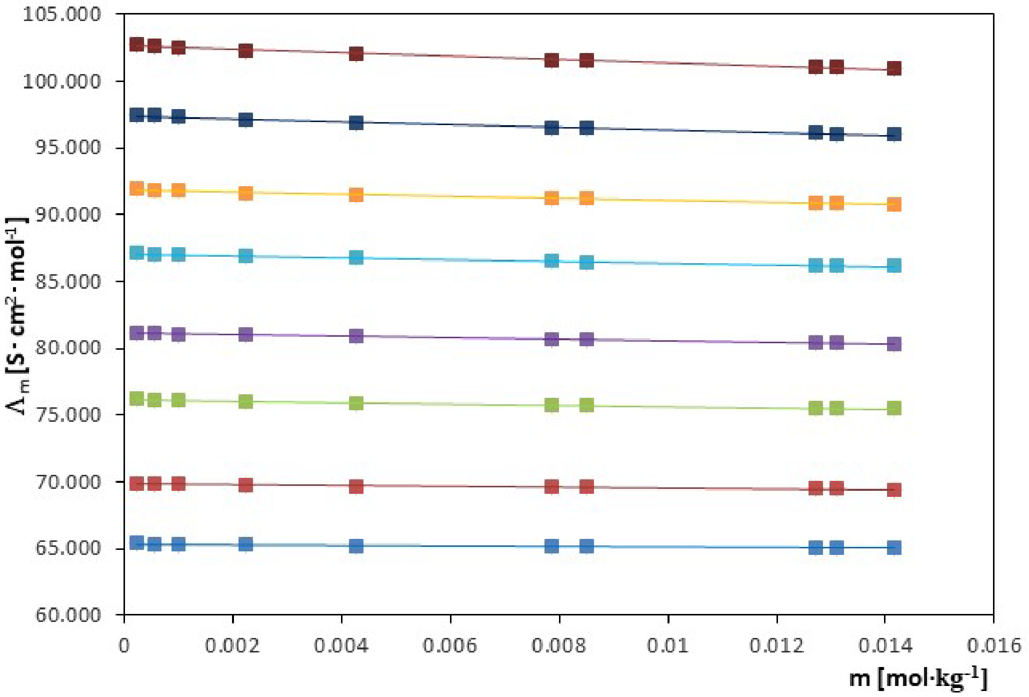

Figure S2. Temperature dependence of molar conductances, Λ_m / $S{\cdot}cm^2{\cdot}mol^{-1}$ for *[emim][Cl] in* N,N-Dimethylformamide for : (■) 278.15K, (■) 283.15K, (■) 288.15K (■) 293.15.15K (■) 298.15K, (■) 303.15K, (■) 308.15K and (■) 313.15K

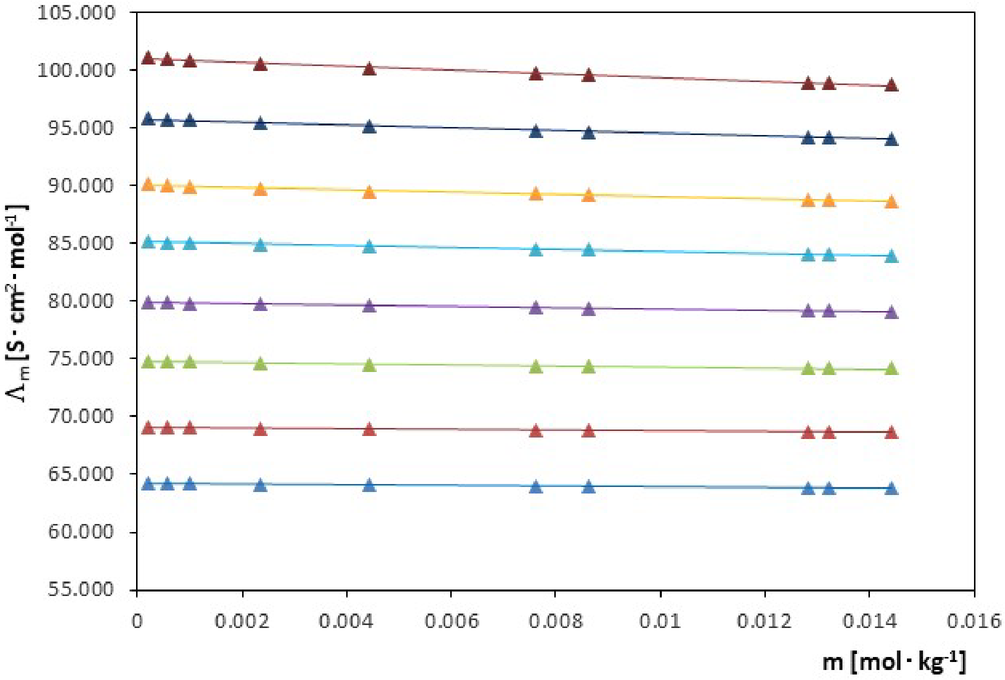

Figure S3. Temperature dependence of molar conductances, Λ_m / $S{\cdot}cm^2{\cdot}mol^{-1}$ for *[bmim][Cl] in* N,N-Dimethylformamide for: (▲) 278.15K, (▲) 283.15K, (▲) 288.15K (▲) 293.15.15K (▲) 298.15K, (▲) 303.15K, (▲) 308.15K and (▲) 13.15K

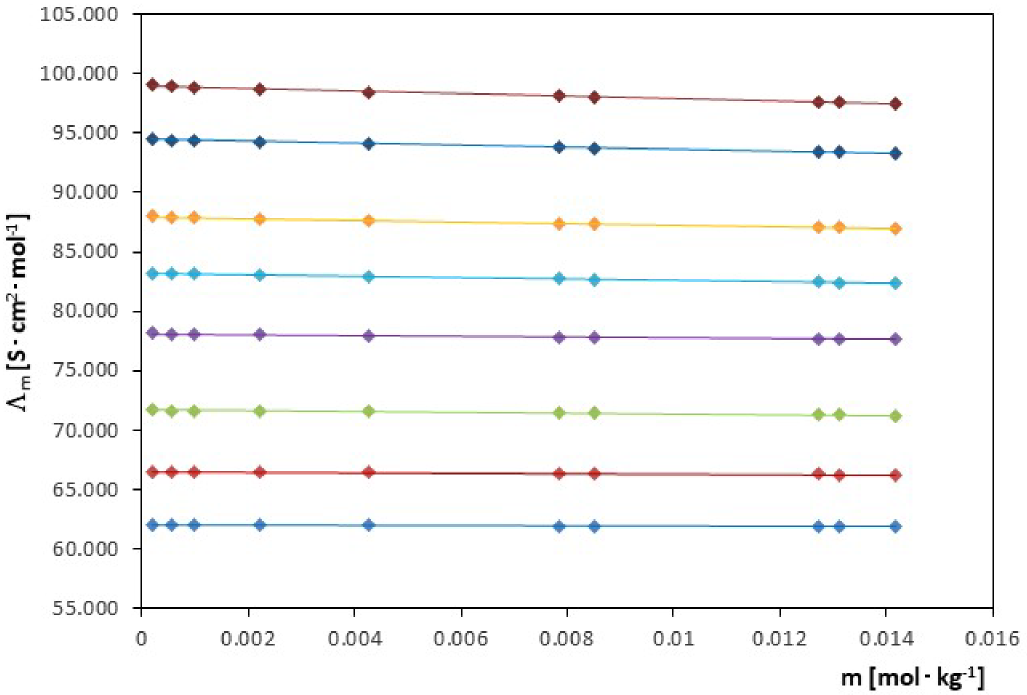

Figure S4. Temperature dependence of molar conductances, Λ_m / $S{\cdot}cm^2{\cdot}mol^{-1}$ for *[hmim][Cl] in* N,N-Dimethylformamide for : (♦) 278.15K, (♦) 283.15K, (♦) 288.15K (♦) 293.15.15K (♦) 298.15K, (♦) 303.15K, (♦) 308.15K and (♦) 313.15K K

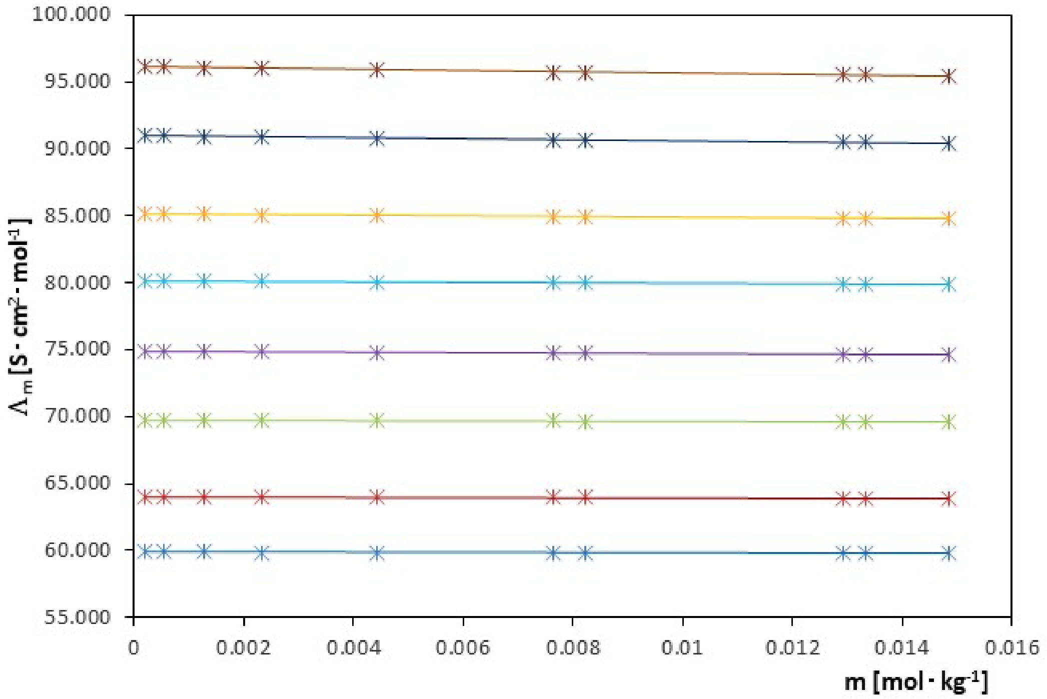

Figure S5. Temperature dependence of molar conductances, Λ_m / $S{\cdot}cm^2{\cdot}mol^{-1}$ for *[omim][Cl] in* N,N-Dimethylformamide for : (✳) 278.15K, (✳) 283.15K, (✳) 288.15K (✳) 293.15.15K (✳) 298.15K, (✳) 303.15K, (✳) 308.15K and (✳) 313.15K.
